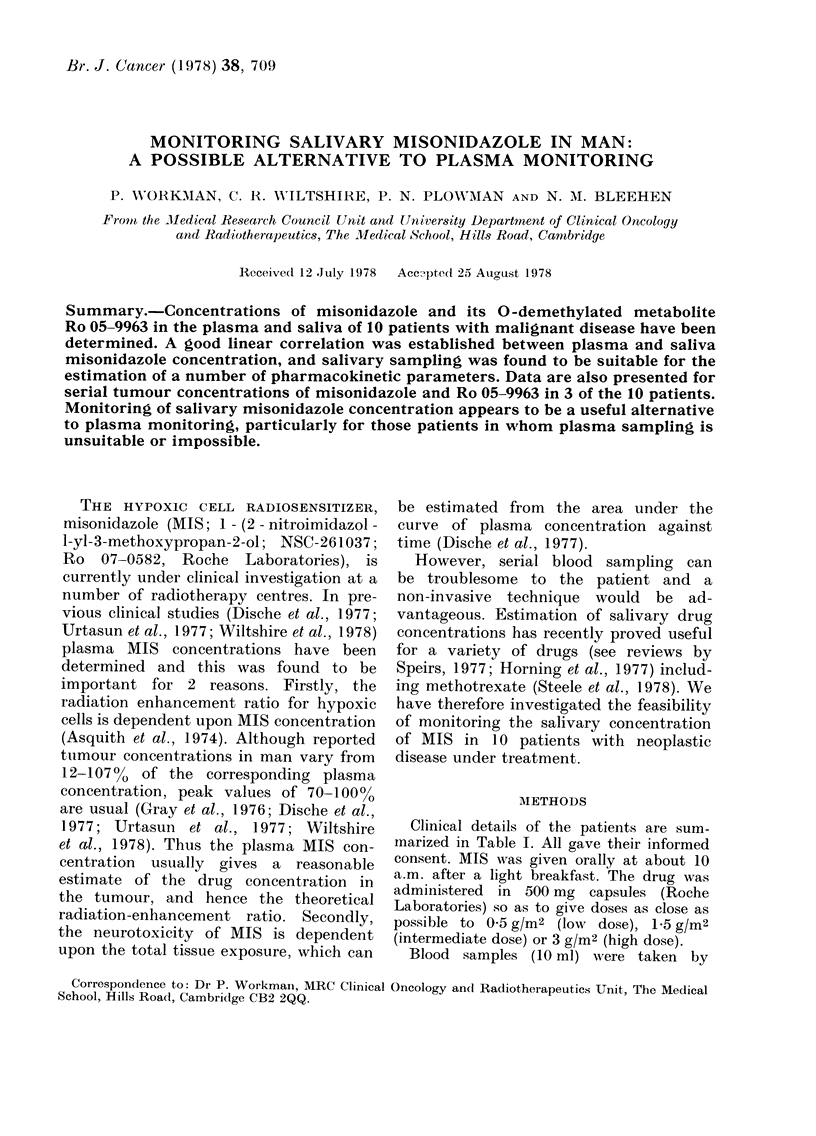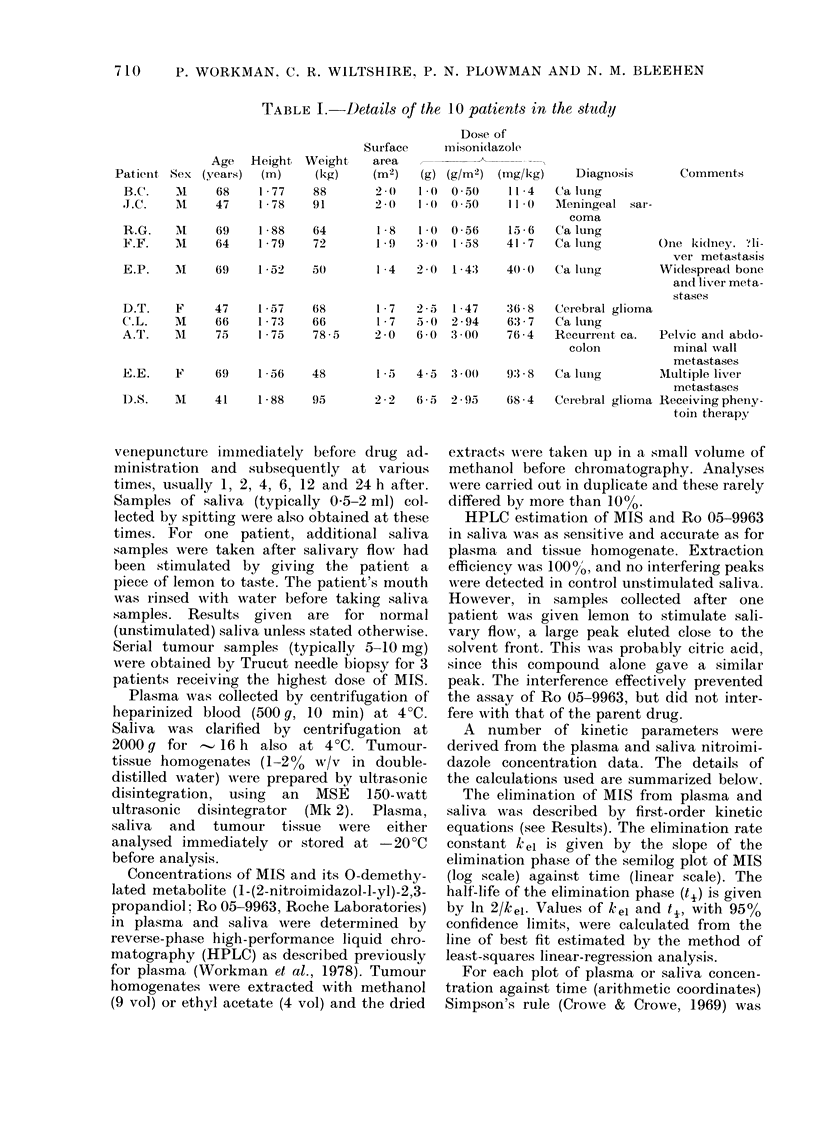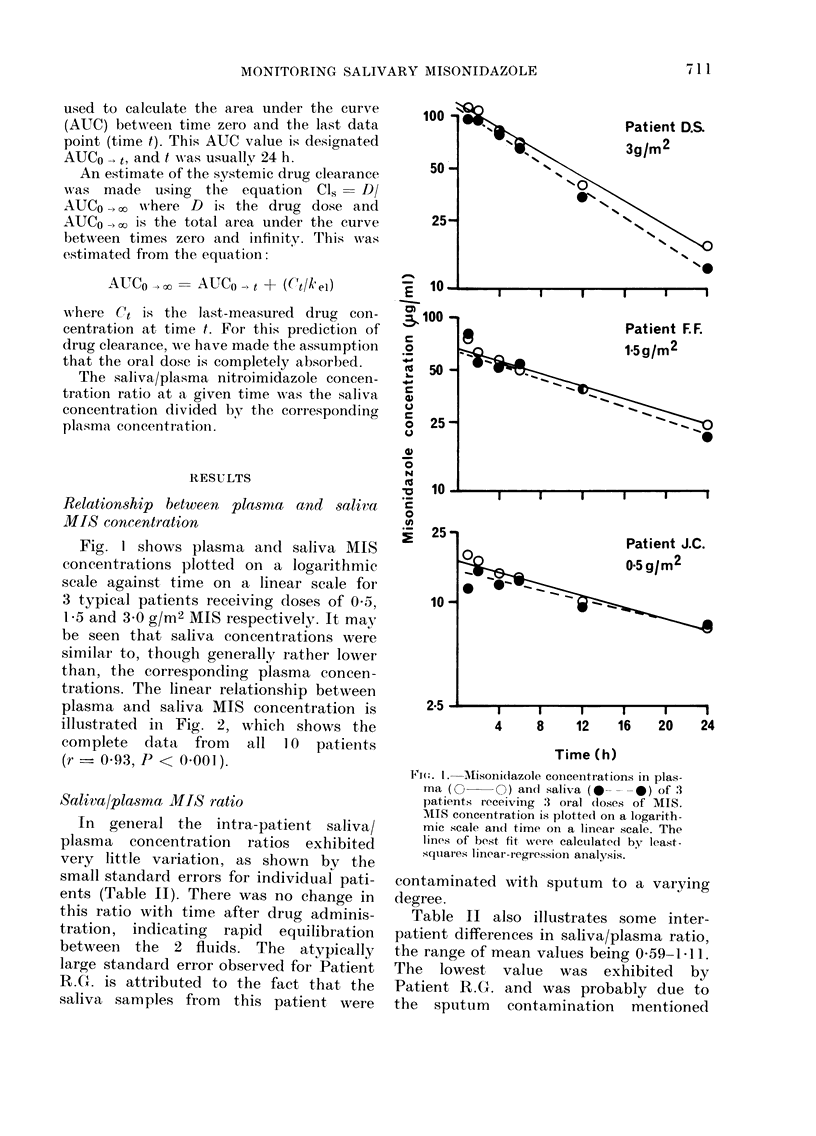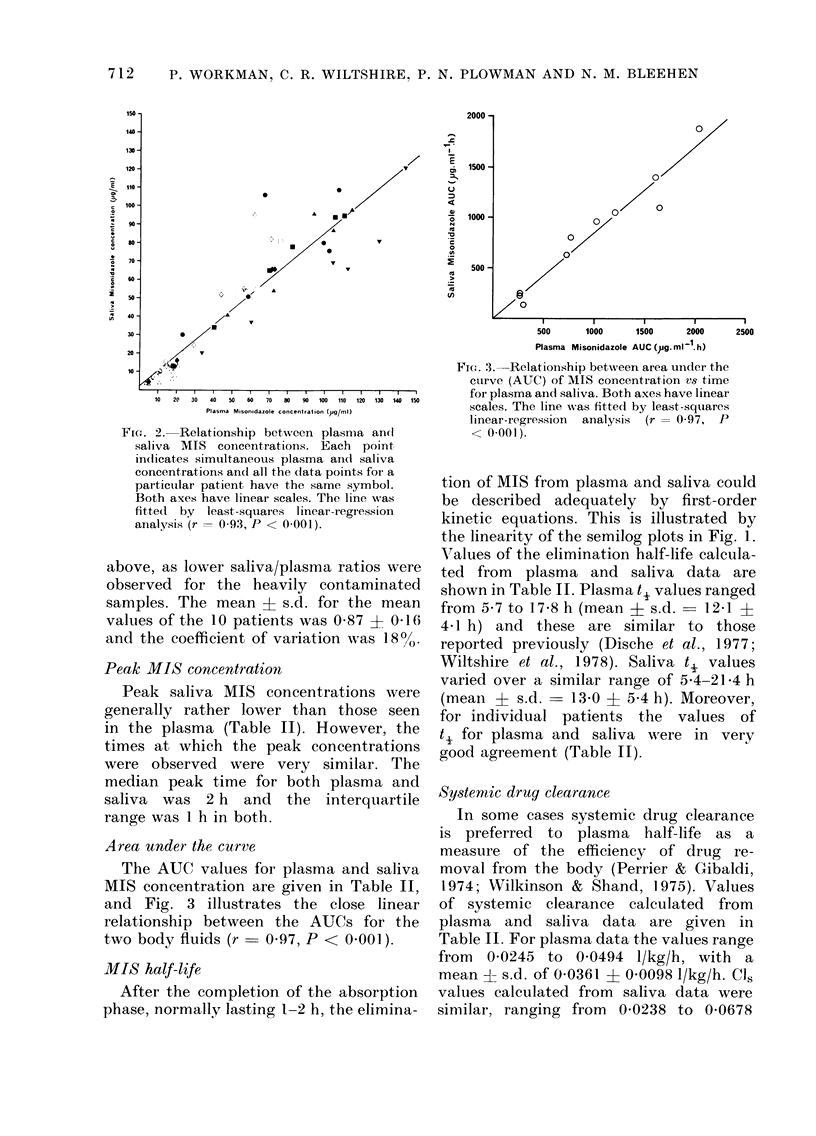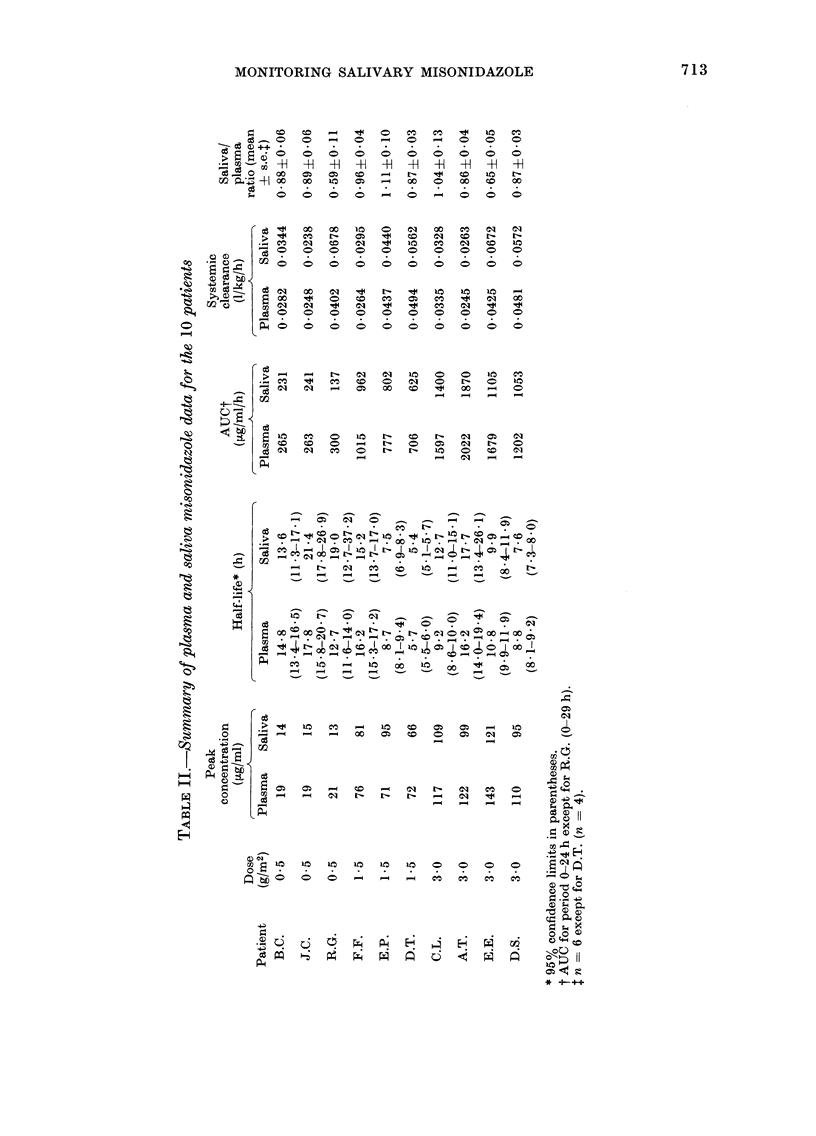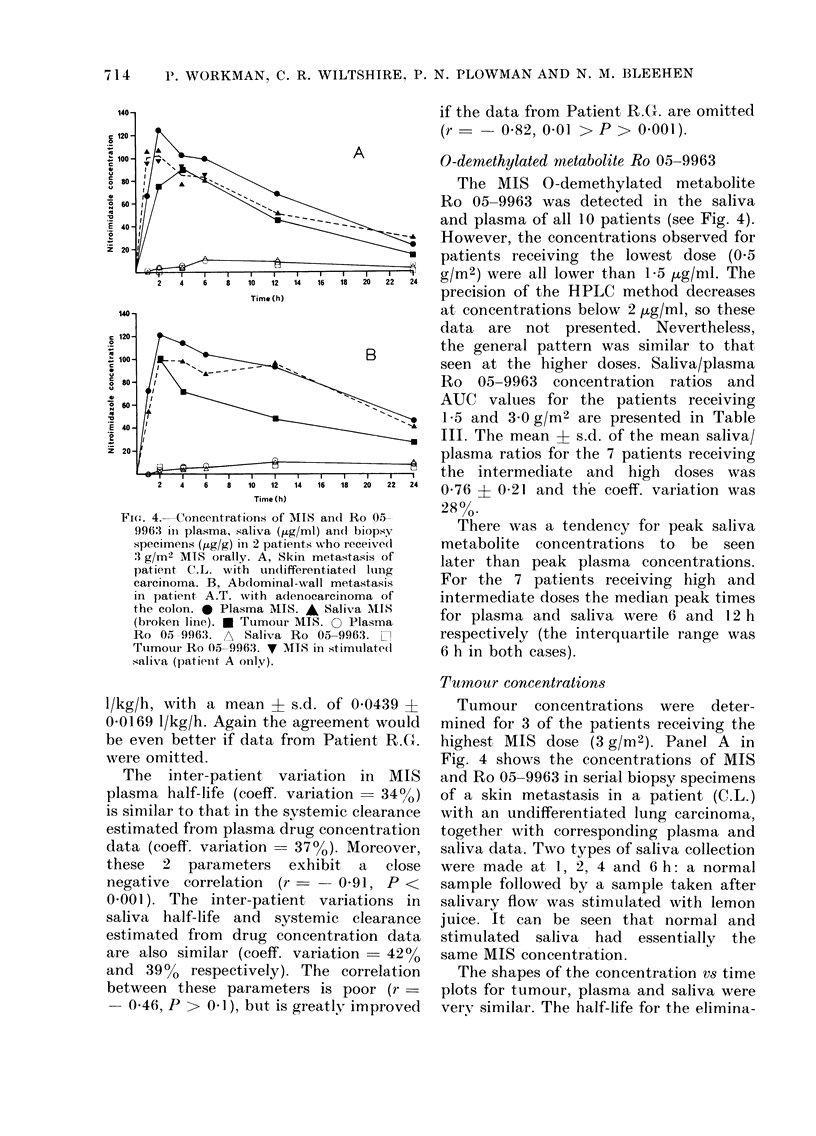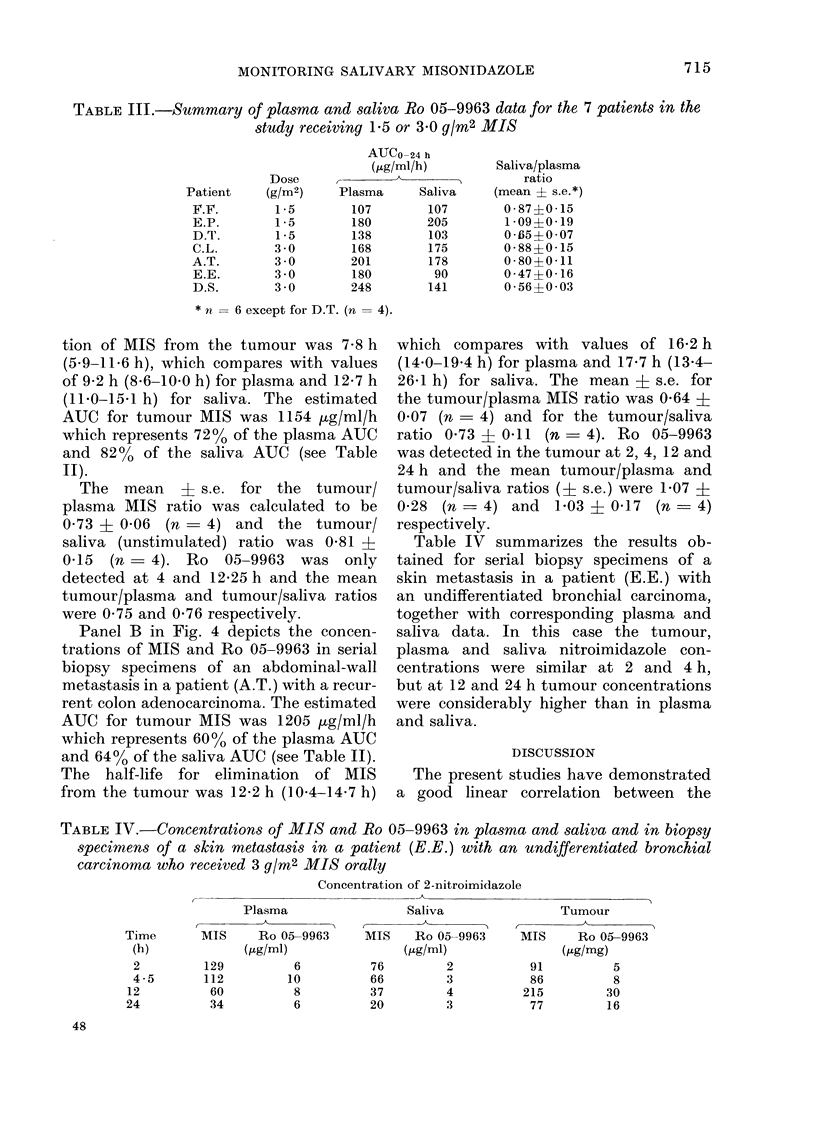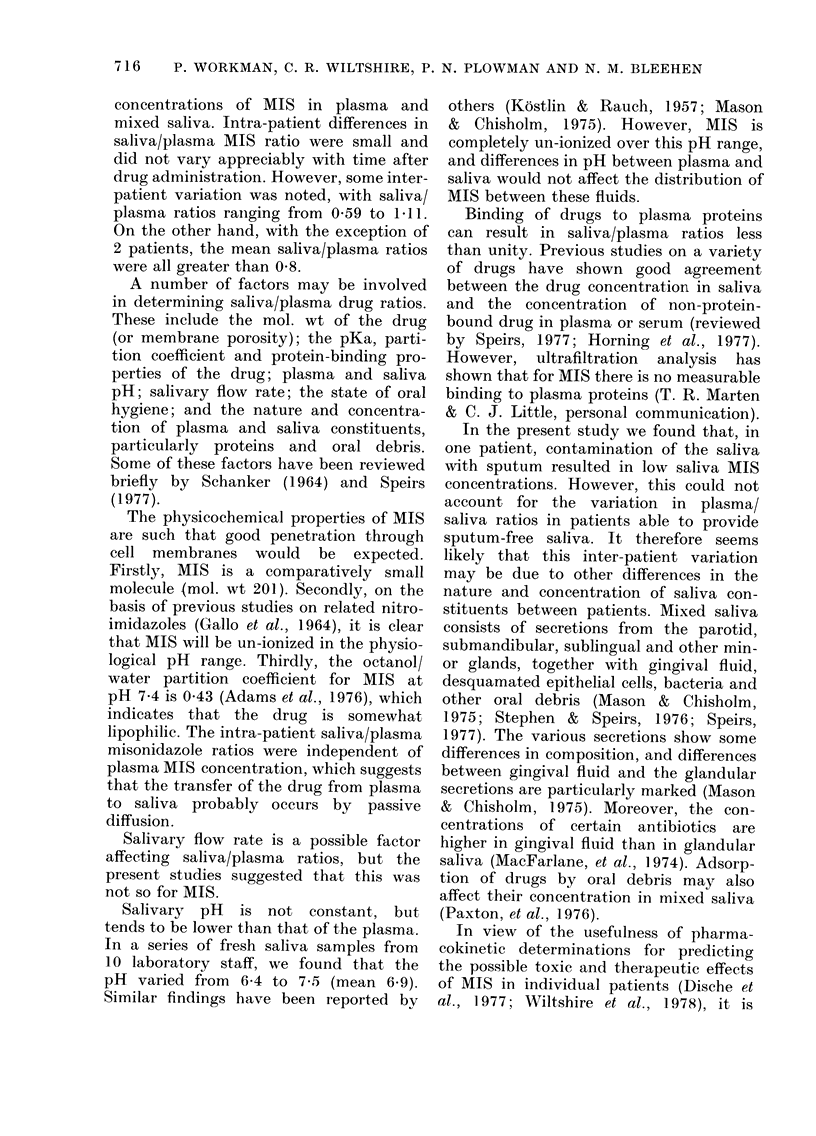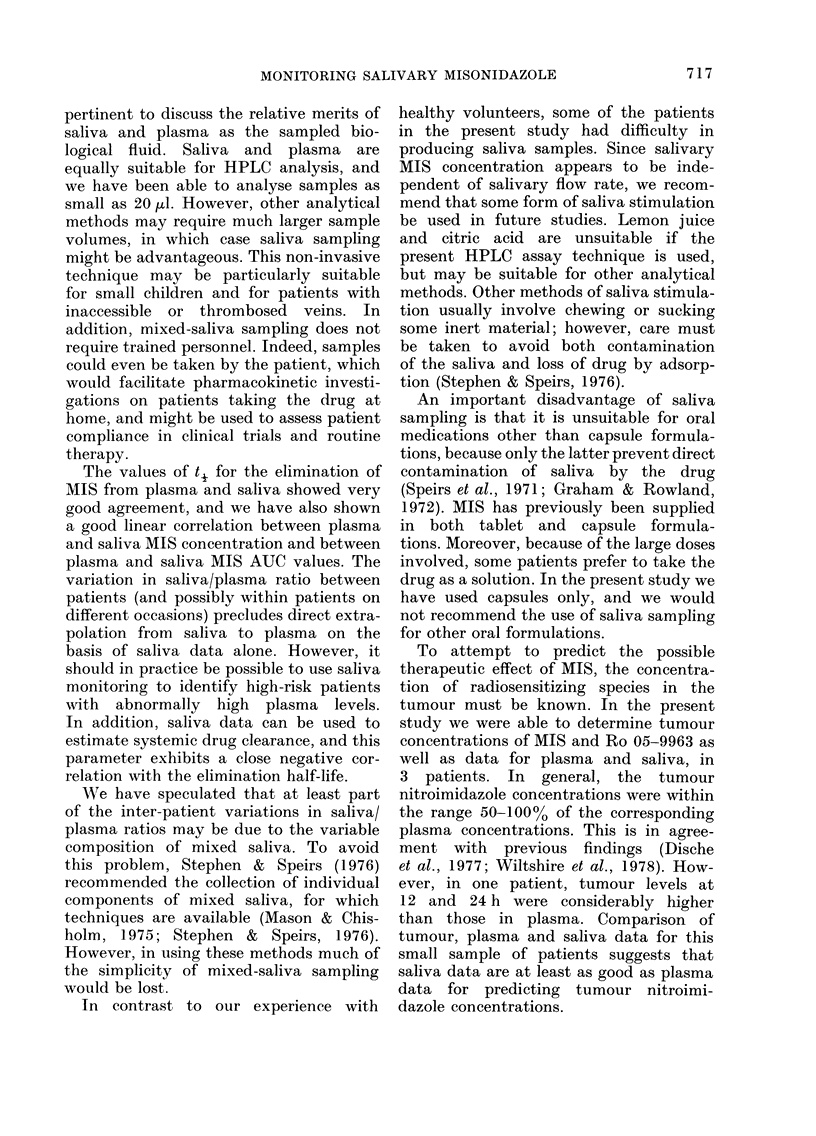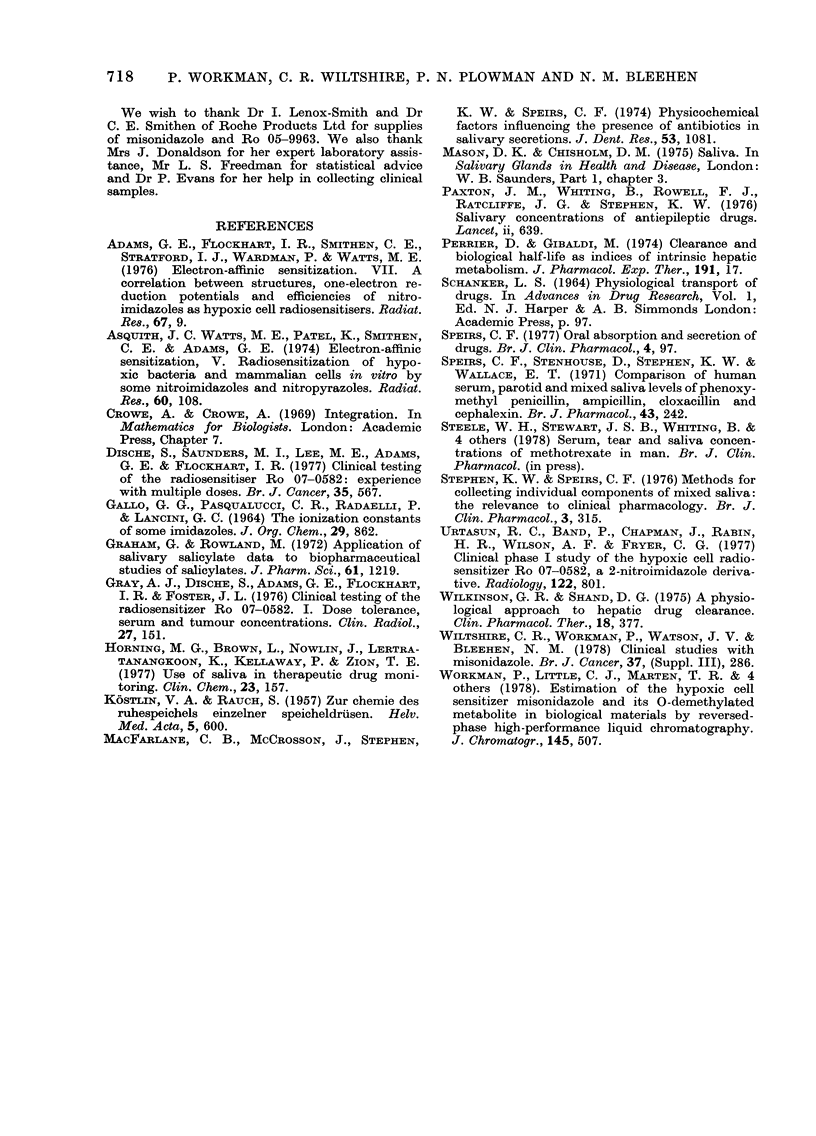# Monitoring salivary misonidazole in man: a possible alternative to plasma monitoring.

**DOI:** 10.1038/bjc.1978.277

**Published:** 1978-12

**Authors:** P. Workman, C. R. Wiltshire, P. N. Plowman, N. M. Bleehen

## Abstract

Concentrations of misonidazole and its O-demethylated metabolite Ro 05-9963 in the plasma and saliva of 10 patients with malignant disease have been determined. A good linear correlation was established between plasma and saliva misonidazole concentration, and salivary sampling was found to be suitable for the estimation of a number of pharmacokinetic parameters. Data are also presented for serial tumour cencentrations of misonidazole and Ro 05-9963 in 3 of the 10 patients. Monitoring of salivary misonidazole concentration appears to be a useful alternative to plasma monitoring, particularly for those patients in whom plasma sampling is unsuitable or impossible.


*Br. J. Cancer* (1978) **38**, 709

### MONITORING SALIVARY MISONIDAZOLE IN MAN: A POSSIBLE ALTERNATIVE TO PLASMA MONITORING

P. WORKMAN, C. R. WILTSHIRE, P. N. PLOWMAN AND N. M. BLEEHEN

*From the Medical Research Council Unit and University Department of Clinical Oncology and Radiotherapeutics, The Medical School, Hills Road, Cambridge*

Received 12 July 1978 Accepted 25 August 1978

**Summary.—Concentrations of misonidazole and its O-demethylated metabolite Ro 05–9963 in the plasma and saliva of 10 patients with malignant disease have been determined. A good linear correlation was established between plasma and saliva misonidazole concentration, and salivary sampling was found to be suitable for the estimation of a number of pharmacokinetic parameters. Data are also presented for serial tumour concentrations of misonidazole and Ro 05–9963 in 3 of the 10 patients. Monitoring of salivary misonidazole concentration appears to be a useful alternative to plasma monitoring, particularly for those patients in whom plasma sampling is unsuitable or impossible.**

THE HYPOXIC CELL RADIOSENSITIZER, misonidazole (MIS; 1 - (2 - nitroimidazol - l-yl-3-methoxypropan-2-ol; NSC-261037; Ro 07–0582, Roche Laboratories), is currently under clinical investigation at a number of radiotherapy centres. In previous clinical studies (Dische *et al.*, 1977; Urtasun *et al.*, 1977; Wiltshire *et al.*, 1978) plasma MIS concentrations have been determined and this was found to be important for 2 reasons. Firstly, the radiation enhancement ratio for hypoxic cells is dependent upon MIS concentration (Asquith *et al.*, 1974). Although reported tumour concentrations in man vary from 12–107% of the corresponding plasma concentration, peak values of 70–100% are usual (Gray *et al.*, 1976; Dische *et al.*, 1977; Urtasun *et al.*, 1977; Wiltshire *et al.*, 1978). Thus the plasma MIS concentration usually gives a reasonable estimate of the drug concentration in the tumour, and hence the theoretical radiation-enhancement ratio. Secondly, the neurotoxicity of MIS is dependent upon the total tissue exposure, which can be estimated from the area under the curve of plasma concentration against time (Dische *et al.*, 1977).

However, serial blood sampling can be troublesome to the patient and a non-invasive technique would be advantageous. Estimation of salivary drug concentrations has recently proved useful for a variety of drugs (see reviews by Speirs, 1977; Horning *et al.*, 1977) including methotrexate (Steele *et al.*, 1978). We have therefore investigated the feasibility of monitoring the salivary concentration of MIS in 10 patients with neoplastic disease under treatment.

### METHODS

Clinical details of the patients are summarized in Table I. All gave their informed consent. MIS was given orally at about 10 a.m. after a light breakfast. The drug was administered in 500 mg capsules (Roche Laboratories) so as to give doses as close as possible to 0·5 g/m² (low dose), 1·5 g/m² (intermediate dose) or 3 g/m² (high dose).

Blood samples (10 ml) were taken by

Correspondence to: Dr P. Workman, MRC Clinical Oncology and Radiotherapeutics Unit, The Medical School, Hills Road, Cambridge CB2 2QQ.

TABLE I.—*Details of the* 10 *patients in the study*

| Patient | Sex | Age (years) | Height (m) | Weight (kg) | Surface area ($m^2$) | Dose of misonidazole (g) | ($g/m^2$) | (mg/kg) | Diagnosis | Comments |
|---|---|---|---|---|---|---|---|---|---|---|
| B.C. | M | 68 | 1·77 | 88 | 2·0 | 1·0 | 0·50 | 11·4 | Ca lung | |
| J.C. | M | 47 | 1·78 | 91 | 2·0 | 1·0 | 0·50 | 11·0 | Meningeal sarcoma | |
| R.G. | M | 69 | 1·88 | 64 | 1·8 | 1·0 | 0·56 | 15·6 | Ca lung | |
| F.F. | M | 64 | 1·79 | 72 | 1·9 | 3·0 | 1·58 | 41·7 | Ca lung | One kidney. ?liver metastasis |
| E.P. | M | 69 | 1·52 | 50 | 1·4 | 2·0 | 1·43 | 40·0 | Ca lung | Widespread bone and liver metastases |
| D.T. | F | 47 | 1·57 | 68 | 1·7 | 2·5 | 1·47 | 36·8 | Cerebral glioma | |
| C.L. | M | 66 | 1·73 | 66 | 1·7 | 5·0 | 2·94 | 63·7 | Ca lung | |
| A.T. | M | 75 | 1·75 | 78·5 | 2·0 | 6·0 | 3·00 | 76·4 | Recurrent ca. colon | Pelvic and abdominal wall metastases |
| E.E. | F | 69 | 1·56 | 48 | 1·5 | 4·5 | 3·00 | 93·8 | Ca lung | Multiple liver metastases |
| D.S. | M | 41 | 1·88 | 95 | 2·2 | 6·5 | 2·95 | 68·4 | Cerebral glioma | Receiving phenytoin therapy |

venepuncture immediately before drug administration and subsequently at various times, usually 1, 2, 4, 6, 12 and 24 h after. Samples of saliva (typically 0·5–2 ml) collected by spitting were also obtained at these times. For one patient, additional saliva samples were taken after salivary flow had been stimulated by giving the patient a piece of lemon to taste. The patient's mouth was rinsed with water before taking saliva samples. Results given are for normal (unstimulated) saliva unless stated otherwise. Serial tumour samples (typically 5–10 mg) were obtained by Trucut needle biopsy for 3 patients receiving the highest dose of MIS.

Plasma was collected by centrifugation of heparinized blood (500 *g*, 10 min) at 4°C. Saliva was clarified by centrifugation at 2000 *g* for ~16 h also at 4°C. Tumour-tissue homogenates (1–2% w/v in double-distilled water) were prepared by ultrasonic disintegration, using an MSE 150-watt ultrasonic disintegrator (Mk 2). Plasma, saliva and tumour tissue were either analysed immediately or stored at −20°C before analysis.

Concentrations of MIS and its O-demethylated metabolite (1-(2-nitroimidazol-l-yl)-2,3-propandiol; Ro 05–9963, Roche Laboratories) in plasma and saliva were determined by reverse-phase high-performance liquid chromatography (HPLC) as described previously for plasma (Workman *et al.*, 1978). Tumour homogenates were extracted with methanol (9 vol) or ethyl acetate (4 vol) and the dried extracts were taken up in a small volume of methanol before chromatography. Analyses were carried out in duplicate and these rarely differed by more than 10%.

HPLC estimation of MIS and Ro 05–9963 in saliva was as sensitive and accurate as for plasma and tissue homogenate. Extraction efficiency was 100%, and no interfering peaks were detected in control unstimulated saliva. However, in samples collected after one patient was given lemon to stimulate salivary flow, a large peak eluted close to the solvent front. This was probably citric acid, since this compound alone gave a similar peak. The interference effectively prevented the assay of Ro 05–9963, but did not interfere with that of the parent drug.

A number of kinetic parameters were derived from the plasma and saliva nitroimidazole concentration data. The details of the calculations used are summarized below.

The elimination of MIS from plasma and saliva was described by first-order kinetic equations (see Results). The elimination rate constant $k_{el}$ is given by the slope of the elimination phase of the semilog plot of MIS (log scale) against time (linear scale). The half-life of the elimination phase ($t_{\frac{1}{2}}$) is given by ln $2/k_{el}$. Values of $k_{el}$ and $t_{\frac{1}{2}}$, with 95% confidence limits, were calculated from the line of best fit estimated by the method of least-squares linear-regression analysis.

For each plot of plasma or saliva concentration against time (arithmetic coordinates) Simpson's rule (Crowe & Crowe, 1969) was used to calculate the area under the curve (AUC) between time zero and the last data point (time $t$). This AUC value is designated $AUC_{0 \to t}$, and $t$ was usually 24 h.

An estimate of the systemic drug clearance was made using the equation $Cl_s = D/AUC_{0 \to \infty}$ where $D$ is the drug dose and $AUC_{0 \to \infty}$ is the total area under the curve between times zero and infinity. This was estimated from the equation:

$$AUC_{0 \to \infty} = AUC_{0 \to t} + (C_t/k_{el})$$

where $C_t$ is the last-measured drug concentration at time $t$. For this prediction of drug clearance, we have made the assumption that the oral dose is completely absorbed.

The saliva/plasma nitroimidazole concentration ratio at a given time was the saliva concentration divided by the corresponding plasma concentration.

### RESULTS

### *Relationship between plasma and saliva MIS concentration*

Fig. 1 shows plasma and saliva MIS concentrations plotted on a logarithmic scale against time on a linear scale for 3 typical patients receiving doses of 0·5, 1·5 and 3·0 g/m² MIS respectively. It may be seen that saliva concentrations were similar to, though generally rather lower than, the corresponding plasma concentrations. The linear relationship between plasma and saliva MIS concentration is illustrated in Fig. 2, which shows the complete data from all 10 patients ($r = 0{\cdot}93$, $P < 0{\cdot}001$).

### *Saliva/plasma MIS ratio*

In general the intra-patient saliva/plasma concentration ratios exhibited very little variation, as shown by the small standard errors for individual patients (Table II). There was no change in this ratio with time after drug administration, indicating rapid equilibration between the 2 fluids. The atypically large standard error observed for Patient R.G. is attributed to the fact that the saliva samples from this patient were contaminated with sputum to a varying degree.

FIG. 1.—Misonidazole concentrations in plasma (○——○) and saliva (●– – –●) of 3 patients receiving 3 oral doses of MIS. MIS concentration is plotted on a logarithmic scale and time on a linear scale. The lines of best fit were calculated by least-squares linear-regression analysis.

Table II also illustrates some inter-patient differences in saliva/plasma ratio, the range of mean values being 0·59–1·11. The lowest value was exhibited by Patient R.G. and was probably due to the sputum contamination mentioned

FIG. 2.—Relationship between plasma and saliva MIS concentrations. Each point indicates simultaneous plasma and saliva concentrations and all the data points for a particular patient have the same symbol. Both axes have linear scales. The line was fitted by least-squares linear-regression analysis ($r = 0{\cdot}93$, $P < 0{\cdot}001$).

FIG. 3.—Relationship between area under the curve (AUC) of MIS concentration *vs* time for plasma and saliva. Both axes have linear scales. The line was fitted by least-squares linear-regression analysis ($r = 0{\cdot}97$, $P < 0{\cdot}001$).

above, as lower saliva/plasma ratios were observed for the heavily contaminated samples. The mean $\pm$ s.d. for the mean values of the 10 patients was 0·87 $\pm$ 0·16 and the coefficient of variation was 18%.

### *Peak MIS concentration*

Peak saliva MIS concentrations were generally rather lower than those seen in the plasma (Table II). However, the times at which the peak concentrations were observed were very similar. The median peak time for both plasma and saliva was 2 h and the interquartile range was 1 h in both.

### *Area under the curve*

The AUC values for plasma and saliva MIS concentration are given in Table II, and Fig. 3 illustrates the close linear relationship between the AUCs for the two body fluids ($r = 0{\cdot}97$, $P < 0{\cdot}001$).

### *MIS half-life*

After the completion of the absorption phase, normally lasting 1–2 h, the elimination of MIS from plasma and saliva could be described adequately by first-order kinetic equations. This is illustrated by the linearity of the semilog plots in Fig. 1. Values of the elimination half-life calculated from plasma and saliva data are shown in Table II. Plasma $t_{\frac{1}{2}}$ values ranged from 5·7 to 17·8 h (mean $\pm$ s.d. = 12·1 $\pm$ 4·1 h) and these are similar to those reported previously (Dische *et al.*, 1977; Wiltshire *et al.*, 1978). Saliva $t_{\frac{1}{2}}$ values varied over a similar range of 5·4–21·4 h (mean $\pm$ s.d. = 13·0 $\pm$ 5·4 h). Moreover, for individual patients the values of $t_{\frac{1}{2}}$ for plasma and saliva were in very good agreement (Table II).

### *Systemic drug clearance*

In some cases systemic drug clearance is preferred to plasma half-life as a measure of the efficiency of drug removal from the body (Perrier & Gibaldi, 1974; Wilkinson & Shand, 1975). Values of systemic clearance calculated from plasma and saliva data are given in Table II. For plasma data the values range from 0·0245 to 0·0494 l/kg/h, with a mean $\pm$ s.d. of 0·0361 $\pm$ 0·0098 l/kg/h. $Cl_s$ values calculated from saliva data were similar, ranging from 0·0238 to 0·0678

TABLE II.—*Summary of plasma and saliva misonidazole data for the* 10 *patients*

| Patient | Dose (g/m²) | Peak concentration (μg/ml) Plasma | Peak concentration (μg/ml) Saliva | Half-life* (h) Plasma | Half-life* (h) Saliva | AUC† (μg/ml/h) Plasma | AUC† (μg/ml/h) Saliva | Systemic clearance (l/kg/h) Plasma | Systemic clearance (l/kg/h) Saliva | Saliva/plasma ratio (mean ± s.e.‡) |
|---|---|---|---|---|---|---|---|---|---|---|
| B.C. | 0·5 | 19 | 14 | 14·8 (13·4–16·5) | 13·6 (11·3–17·1) | 265 | 231 | 0·0282 | 0·0344 | 0·88±0·06 |
| J.C. | 0·5 | 19 | 15 | 17·8 (15·8–20·7) | 21·4 (17·8–26·9) | 263 | 241 | 0·0248 | 0·0238 | 0·89±0·06 |
| R.G. | 0·5 | 21 | 13 | 12·7 (11·6–14·0) | 19·0 (12·7–37·2) | 300 | 137 | 0·0402 | 0·0678 | 0·59±0·11 |
| F.F. | 1·5 | 76 | 81 | 16·2 (15·3–17·2) | 15·2 (13·7–17·0) | 1015 | 962 | 0·0264 | 0·0295 | 0·96±0·04 |
| E.P. | 1·5 | 71 | 95 | 8·7 (8·1–9·4) | 7·5 (6·9–8·3) | 777 | 802 | 0·0437 | 0·0440 | 1·11±0·10 |
| D.T. | 1·5 | 72 | 66 | 5·7 (5·5–6·0) | 5·4 (5·1–5·7) | 706 | 625 | 0·0494 | 0·0562 | 0·87±0·03 |
| C.L. | 3·0 | 117 | 109 | 9·2 (8·6–10·0) | 12·7 (11·0–15·1) | 1597 | 1400 | 0·0335 | 0·0328 | 1·04±0·13 |
| A.T. | 3·0 | 122 | 99 | 16·2 (14·0–19·4) | 17·7 (13·4–26·1) | 2022 | 1870 | 0·0245 | 0·0263 | 0·86±0·04 |
| E.E. | 3·0 | 143 | 121 | 10·8 (9·9–11·9) | 9·9 (8·4–11·9) | 1679 | 1105 | 0·0425 | 0·0672 | 0·65±0·05 |
| D.S. | 3·0 | 110 | 95 | 8·8 (8·1–9·2) | 7·6 (7·3–8·0) | 1202 | 1053 | 0·0481 | 0·0572 | 0·87±0·03 |

* 95% confidence limits in parentheses.

† AUC for period 0–24 h except for R.G. (0–29 h).

‡ $n = 6$ except for D.T. ($n = 4$).

FIG. 4.—Concentrations of MIS and Ro 05–9963 in plasma, saliva (μg/ml) and biopsy specimens (μg/g) in 2 patients who received 3 g/m$^2$ MIS orally. A, Skin metastasis of patient C.L. with undifferentiated lung carcinoma. B, Abdominal-wall metastasis in patient A.T. with adenocarcinoma of the colon. ● Plasma MIS. ▲ Saliva MIS (broken line). ■ Tumour MIS. ○ Plasma Ro 05–9963. △ Saliva Ro 05–9963. □ Tumour Ro 05–9963. ▼ MIS in stimulated saliva (patient A only).

l/kg/h, with a mean ± s.d. of 0·0439 ± 0·0169 l/kg/h. Again the agreement would be even better if data from Patient R.G. were omitted.

The inter-patient variation in MIS plasma half-life (coeff. variation = 34%) is similar to that in the systemic clearance estimated from plasma drug concentration data (coeff. variation = 37%). Moreover, these 2 parameters exhibit a close negative correlation ($r = -0{\cdot}91$, $P < 0{\cdot}001$). The inter-patient variations in saliva half-life and systemic clearance estimated from drug concentration data are also similar (coeff. variation = 42% and 39% respectively). The correlation between these parameters is poor ($r = -0{\cdot}46$, $P > 0{\cdot}1$), but is greatly improved if the data from Patient R.G. are omitted ($r = -0{\cdot}82$, $0{\cdot}01 > P > 0{\cdot}001$).

*O-demethylated metabolite Ro* 05–9963

The MIS O-demethylated metabolite Ro 05–9963 was detected in the saliva and plasma of all 10 patients (see Fig. 4). However, the concentrations observed for patients receiving the lowest dose (0·5 g/m$^2$) were all lower than 1·5 μg/ml. The precision of the HPLC method decreases at concentrations below 2 μg/ml, so these data are not presented. Nevertheless, the general pattern was similar to that seen at the higher doses. Saliva/plasma Ro 05–9963 concentration ratios and AUC values for the patients receiving 1·5 and 3·0 g/m$^2$ are presented in Table III. The mean ± s.d. of the mean saliva/plasma ratios for the 7 patients receiving the intermediate and high doses was 0·76 ± 0·21 and the coeff. variation was 28%.

There was a tendency for peak saliva metabolite concentrations to be seen later than peak plasma concentrations. For the 7 patients receiving high and intermediate doses the median peak times for plasma and saliva were 6 and 12 h respectively (the interquartile range was 6 h in both cases).

*Tumour concentrations*

Tumour concentrations were determined for 3 of the patients receiving the highest MIS dose (3 g/m$^2$). Panel A in Fig. 4 shows the concentrations of MIS and Ro 05–9963 in serial biopsy specimens of a skin metastasis in a patient (C.L.) with an undifferentiated lung carcinoma, together with corresponding plasma and saliva data. Two types of saliva collection were made at 1, 2, 4 and 6 h: a normal sample followed by a sample taken after salivary flow was stimulated with lemon juice. It can be seen that normal and stimulated saliva had essentially the same MIS concentration.

The shapes of the concentration *vs* time plots for tumour, plasma and saliva were very similar. The half-life for the elimina-

TABLE III.—*Summary of plasma and saliva Ro* 05–9963 *data for the* 7 *patients in the study receiving* 1·5 *or* 3·0 *g/m² MIS*

| Patient | Dose ($g/m^2$) | $AUC_{0-24\ h}$ ($\mu g/ml/h$) Plasma | Saliva | Saliva/plasma ratio (mean ± s.e.*) |
|---|---|---|---|---|
| F.F. | 1·5 | 107 | 107 | 0·87±0·15 |
| E.P. | 1·5 | 180 | 205 | 1·09±0·19 |
| D.T. | 1·5 | 138 | 103 | 0·65±0·07 |
| C.L. | 3·0 | 168 | 175 | 0·88±0·15 |
| A.T. | 3·0 | 201 | 178 | 0·80±0·11 |
| E.E. | 3·0 | 180 | 90 | 0·47±0·16 |
| D.S. | 3·0 | 248 | 141 | 0·56±0·03 |

* $n$ = 6 except for D.T. ($n$ = 4).

tion of MIS from the tumour was 7·8 h (5·9–11·6 h), which compares with values of 9·2 h (8·6–10·0 h) for plasma and 12·7 h (11·0–15·1 h) for saliva. The estimated AUC for tumour MIS was 1154 μg/ml/h which represents 72% of the plasma AUC and 82% of the saliva AUC (see Table II).

The mean ± s.e. for the tumour/plasma MIS ratio was calculated to be 0·73 ± 0·06 ($n$ = 4) and the tumour/saliva (unstimulated) ratio was 0·81 ± 0·15 ($n$ = 4). Ro 05–9963 was only detected at 4 and 12·25 h and the mean tumour/plasma and tumour/saliva ratios were 0·75 and 0·76 respectively.

Panel B in Fig. 4 depicts the concentrations of MIS and Ro 05–9963 in serial biopsy specimens of an abdominal-wall metastasis in a patient (A.T.) with a recurrent colon adenocarcinoma. The estimated AUC for tumour MIS was 1205 μg/ml/h which represents 60% of the plasma AUC and 64% of the saliva AUC (see Table II). The half-life for elimination of MIS from the tumour was 12·2 h (10·4–14·7 h) which compares with values of 16·2 h (14·0–19·4 h) for plasma and 17·7 h (13·4–26·1 h) for saliva. The mean ± s.e. for the tumour/plasma MIS ratio was 0·64 ± 0·07 ($n$ = 4) and for the tumour/saliva ratio 0·73 ± 0·11 ($n$ = 4). Ro 05–9963 was detected in the tumour at 2, 4, 12 and 24 h and the mean tumour/plasma and tumour/saliva ratios (± s.e.) were 1·07 ± 0·28 ($n$ = 4) and 1·03 ± 0·17 ($n$ = 4) respectively.

Table IV summarizes the results obtained for serial biopsy specimens of a skin metastasis in a patient (E.E.) with an undifferentiated bronchial carcinoma, together with corresponding plasma and saliva data. In this case the tumour, plasma and saliva nitroimidazole concentrations were similar at 2 and 4 h, but at 12 and 24 h tumour concentrations were considerably higher than in plasma and saliva.

### DISCUSSION

The present studies have demonstrated a good linear correlation between the

TABLE IV.—*Concentrations of MIS and Ro* 05–9963 *in plasma and saliva and in biopsy specimens of a skin metastasis in a patient* (*E.E.*) *with an undifferentiated bronchial carcinoma who received* 3 *g/m² MIS orally*

| Time (h) | Concentration of 2-nitroimidazole: Plasma MIS (μg/ml) | Plasma Ro 05–9963 (μg/ml) | Saliva MIS (μg/ml) | Saliva Ro 05–9963 (μg/ml) | Tumour MIS (μg/mg) | Tumour Ro 05–9963 (μg/mg) |
|---|---|---|---|---|---|---|
| 2 | 129 | 6 | 76 | 2 | 91 | 5 |
| 4·5 | 112 | 10 | 66 | 3 | 86 | 8 |
| 12 | 60 | 8 | 37 | 4 | 215 | 30 |
| 24 | 34 | 6 | 20 | 3 | 77 | 16 |

concentrations of MIS in plasma and mixed saliva. Intra-patient differences in saliva/plasma MIS ratio were small and did not vary appreciably with time after drug administration. However, some inter-patient variation was noted, with saliva/plasma ratios ranging from 0·59 to 1·11. On the other hand, with the exception of 2 patients, the mean saliva/plasma ratios were all greater than 0·8.

A number of factors may be involved in determining saliva/plasma drug ratios. These include the mol. wt of the drug (or membrane porosity); the pKa, partition coefficient and protein-binding properties of the drug; plasma and saliva pH; salivary flow rate; the state of oral hygiene; and the nature and concentration of plasma and saliva constituents, particularly proteins and oral debris. Some of these factors have been reviewed briefly by Schanker (1964) and Speirs (1977).

The physicochemical properties of MIS are such that good penetration through cell membranes would be expected. Firstly, MIS is a comparatively small molecule (mol. wt 201). Secondly, on the basis of previous studies on related nitroimidazoles (Gallo *et al.*, 1964), it is clear that MIS will be un-ionized in the physiological pH range. Thirdly, the octanol/water partition coefficient for MIS at pH 7·4 is 0·43 (Adams *et al.*, 1976), which indicates that the drug is somewhat lipophilic. The intra-patient saliva/plasma misonidazole ratios were independent of plasma MIS concentration, which suggests that the transfer of the drug from plasma to saliva probably occurs by passive diffusion.

Salivary flow rate is a possible factor affecting saliva/plasma ratios, but the present studies suggested that this was not so for MIS.

Salivary pH is not constant, but tends to be lower than that of the plasma. In a series of fresh saliva samples from 10 laboratory staff, we found that the pH varied from 6·4 to 7·5 (mean 6·9). Similar findings have been reported by others (Köstlin & Rauch, 1957; Mason & Chisholm, 1975). However, MIS is completely un-ionized over this pH range, and differences in pH between plasma and saliva would not affect the distribution of MIS between these fluids.

Binding of drugs to plasma proteins can result in saliva/plasma ratios less than unity. Previous studies on a variety of drugs have shown good agreement between the drug concentration in saliva and the concentration of non-protein-bound drug in plasma or serum (reviewed by Speirs, 1977; Horning *et al.*, 1977). However, ultrafiltration analysis has shown that for MIS there is no measurable binding to plasma proteins (T. R. Marten & C. J. Little, personal communication).

In the present study we found that, in one patient, contamination of the saliva with sputum resulted in low saliva MIS concentrations. However, this could not account for the variation in plasma/saliva ratios in patients able to provide sputum-free saliva. It therefore seems likely that this inter-patient variation may be due to other differences in the nature and concentration of saliva constituents between patients. Mixed saliva consists of secretions from the parotid, submandibular, sublingual and other minor glands, together with gingival fluid, desquamated epithelial cells, bacteria and other oral debris (Mason & Chisholm, 1975; Stephen & Speirs, 1976; Speirs, 1977). The various secretions show some differences in composition, and differences between gingival fluid and the glandular secretions are particularly marked (Mason & Chisholm, 1975). Moreover, the concentrations of certain antibiotics are higher in gingival fluid than in glandular saliva (MacFarlane, *et al.*, 1974). Adsorption of drugs by oral debris may also affect their concentration in mixed saliva (Paxton, *et al.*, 1976).

In view of the usefulness of pharmacokinetic determinations for predicting the possible toxic and therapeutic effects of MIS in individual patients (Dische *et al.*, 1977; Wiltshire *et al.*, 1978), it is pertinent to discuss the relative merits of saliva and plasma as the sampled biological fluid. Saliva and plasma are equally suitable for HPLC analysis, and we have been able to analyse samples as small as 20 $\mu$l. However, other analytical methods may require much larger sample volumes, in which case saliva sampling might be advantageous. This non-invasive technique may be particularly suitable for small children and for patients with inaccessible or thrombosed veins. In addition, mixed-saliva sampling does not require trained personnel. Indeed, samples could even be taken by the patient, which would facilitate pharmacokinetic investigations on patients taking the drug at home, and might be used to assess patient compliance in clinical trials and routine therapy.

The values of $t_{\frac{1}{2}}$ for the elimination of MIS from plasma and saliva showed very good agreement, and we have also shown a good linear correlation between plasma and saliva MIS concentration and between plasma and saliva MIS AUC values. The variation in saliva/plasma ratio between patients (and possibly within patients on different occasions) precludes direct extrapolation from saliva to plasma on the basis of saliva data alone. However, it should in practice be possible to use saliva monitoring to identify high-risk patients with abnormally high plasma levels. In addition, saliva data can be used to estimate systemic drug clearance, and this parameter exhibits a close negative correlation with the elimination half-life.

We have speculated that at least part of the inter-patient variations in saliva/plasma ratios may be due to the variable composition of mixed saliva. To avoid this problem, Stephen & Speirs (1976) recommended the collection of individual components of mixed saliva, for which techniques are available (Mason & Chisholm, 1975; Stephen & Speirs, 1976). However, in using these methods much of the simplicity of mixed-saliva sampling would be lost.

In contrast to our experience with healthy volunteers, some of the patients in the present study had difficulty in producing saliva samples. Since salivary MIS concentration appears to be independent of salivary flow rate, we recommend that some form of saliva stimulation be used in future studies. Lemon juice and citric acid are unsuitable if the present HPLC assay technique is used, but may be suitable for other analytical methods. Other methods of saliva stimulation usually involve chewing or sucking some inert material; however, care must be taken to avoid both contamination of the saliva and loss of drug by adsorption (Stephen & Speirs, 1976).

An important disadvantage of saliva sampling is that it is unsuitable for oral medications other than capsule formulations, because only the latter prevent direct contamination of saliva by the drug (Speirs *et al.*, 1971; Graham & Rowland, 1972). MIS has previously been supplied in both tablet and capsule formulations. Moreover, because of the large doses involved, some patients prefer to take the drug as a solution. In the present study we have used capsules only, and we would not recommend the use of saliva sampling for other oral formulations.

To attempt to predict the possible therapeutic effect of MIS, the concentration of radiosensitizing species in the tumour must be known. In the present study we were able to determine tumour concentrations of MIS and Ro 05-9963 as well as data for plasma and saliva, in 3 patients. In general, the tumour nitroimidazole concentrations were within the range 50-100% of the corresponding plasma concentrations. This is in agreement with previous findings (Dische *et al.*, 1977; Wiltshire *et al.*, 1978). However, in one patient, tumour levels at 12 and 24 h were considerably higher than those in plasma. Comparison of tumour, plasma and saliva data for this small sample of patients suggests that saliva data are at least as good as plasma data for predicting tumour nitroimidazole concentrations.

We wish to thank Dr I. Lenox-Smith and Dr C. E. Smithen of Roche Products Ltd for supplies of misonidazole and Ro 05–9963. We also thank Mrs J. Donaldson for her expert laboratory assistance, Mr L. S. Freedman for statistical advice and Dr P. Evans for her help in collecting clinical samples.

### REFERENCES

ADAMS, G. E., FLOCKHART, I. R., SMITHEN, C. E., STRATFORD, I. J., WARDMAN, P. & WATTS, M. E. (1976) Electron-affinic sensitization. VII. A correlation between structures, one-electron reduction potentials and efficiencies of nitroimidazoles as hypoxic cell radiosensitisers. *Radiat. Res.*, **67**, 9.

ASQUITH, J. C. WATTS, M. E., PATEL, K., SMITHEN, C. E. & ADAMS, G. E. (1974) Electron-affinic sensitization, V. Radiosensitization of hypoxic bacteria and mammalian cells *in vitro* by some nitroimidazoles and nitropyrazoles. *Radiat. Res.*, **60**, 108.

CROWE, A. & CROWE, A. (1969) Integration. In *Mathematics for Biologists*. London: Academic Press, Chapter 7.

DISCHE, S., SAUNDERS, M. I., LEE, M. E., ADAMS, G. E. & FLOCKHART, I. R. (1977) Clinical testing of the radiosensitiser Ro 07–0582: experience with multiple doses. *Br. J. Cancer*, **35**, 567.

GALLO, G. G., PASQUALUCCI, C. R., RADAELLI, P. & LANCINI, G. C. (1964) The ionization constants of some imidazoles. *J. Org. Chem.*, **29**, 862.

GRAHAM, G. & ROWLAND, M. (1972) Application of salivary salicylate data to biopharmaceutical studies of salicylates. *J. Pharm. Sci.*, **61**, 1219.

GRAY, A. J., DISCHE, S., ADAMS, G. E., FLOCKHART, I. R. & FOSTER, J. L. (1976) Clinical testing of the radiosensitizer Ro 07–0582. I. Dose tolerance, serum and tumour concentrations. *Clin. Radiol.*, **27**, 151.

HORNING, M. G., BROWN, L., NOWLIN, J., LERTRATANANGKOON, K., KELLAWAY, P. & ZION, T. E. (1977) Use of saliva in therapeutic drug monitoring. *Clin. Chem.*, **23**, 157.

KÖSTLIN, V. A. & RAUCH, S. (1957) Zur chemie des ruhespeichels einzelner speicheldrüsen. *Helv. Med. Acta*, **5**, 600.

MACFARLANE, C. B., MCCROSSON, J., STEPHEN, K. W. & SPEIRS, C. F. (1974) Physicochemical factors influencing the presence of antibiotics in salivary secretions. *J. Dent. Res.*, **53**, 1081.

MASON, D. K. & CHISHOLM, D. M. (1975) Saliva. In *Salivary Glands in Health and Disease*, London: W. B. Saunders, Part 1, chapter 3.

PAXTON, J. M., WHITING, B., ROWELL, F. J., RATCLIFFE, J. G. & STEPHEN, K. W. (1976) Salivary concentrations of antiepileptic drugs. *Lancet*, ii, 639.

PERRIER, D. & GIBALDI, M. (1974) Clearance and biological half-life as indices of intrinsic hepatic metabolism. *J. Pharmacol. Exp. Ther.*, **191**, 17.

SCHANKER, L. S. (1964) Physiological transport of drugs. In *Advances in Drug Research*, Vol. 1, Ed. N. J. Harper & A. B. Simmonds London: Academic Press, p. 97.

SPEIRS, C. F. (1977) Oral absorption and secretion of drugs. *Br. J. Clin. Pharmacol.*, **4**, 97.

SPEIRS, C. F., STENHOUSE, D., STEPHEN, K. W. & WALLACE, E. T. (1971) Comparison of human serum, parotid and mixed saliva levels of phenoxymethyl penicillin, ampicillin, cloxacillin and cephalexin. *Br. J. Pharmacol.*, **43**, 242.

STEELE, W. H., STEWART, J. S. B., WHITING, B. & 4 others (1978) Serum, tear and saliva concentrations of methotrexate in man. *Br. J. Clin. Pharmacol.* (in press).

STEPHEN, K. W. & SPEIRS, C. F. (1976) Methods for collecting individual components of mixed saliva: the relevance to clinical pharmacology. *Br. J. Clin. Pharmacol.*, **3**, 315.

URTASUN, R. C., BAND, P., CHAPMAN, J., RABIN, H. R., WILSON, A. F. & FRYER, C. G. (1977) Clinical phase I study of the hypoxic cell radiosensitizer Ro 07–0582, a 2-nitroimidazole derivative. *Radiology*, **122**, 801.

WILKINSON, G. R. & SHAND, D. G. (1975) A physiological approach to hepatic drug clearance. *Clin. Pharmacol. Ther.*, **18**, 377.

WILTSHIRE, C. R., WORKMAN, P., WATSON, J. V. & BLEEHEN, N. M. (1978) Clinical studies with misonidazole. *Br. J. Cancer*, **37**, (Suppl. III), 286.

WORKMAN, P., LITTLE, C. J., MARTEN, T. R. & 4 others (1978). Estimation of the hypoxic cell sensitizer misonidazole and its O-demethylated metabolite in biological materials by reversed-phase high-performance liquid chromatography. *J. Chromatogr.*, **145**, 507.